# Midday Napping and Successful Aging in Older People Living in the Mediterranean Region: The Epidemiological Mediterranean Islands Study (MEDIS)

**DOI:** 10.3390/brainsci10010014

**Published:** 2019-12-26

**Authors:** Alexandra Foscolou, Nathan M. D’Cunha, Nenad Naumovski, Stefanos Tyrovolas, Loukianos Rallidis, Antonia-Leda Matalas, Evangelos Polychronopoulos, Labros S. Sidossis, Demosthenes Panagiotakos

**Affiliations:** 1 Department of Nutrition and Dietetics, School of Health Science and Education, Harokopio University, 17671 Kallithea, Attica, Greece; alexandra.foscolou@gmail.com (A.F.); s.tyrovolas@pssjd.org (S.T.); amatala@hua.gr (A.-L.M.); evpol@hua.gr (E.P.);; 2 Faculty of Health, University of Canberra, Bruce 2617, AustraliaNenad.Naumovski@canberra.edu.au (N.N.); 3 Parc Sanitari Sant Joan de Déu, Fundació Sant Joan de Déu, CIBERSAM, Universitat de Barcelona, 08007 Barcelona, Spain; 4 Second Cardiology Clinic, School of Medicine, University of Athens, 10679 Athens, Greece; lrallidis@gmail.com; 5 Department of Kinesiology and Health, School of Arts and Sciences, Rutgers University, New Brunswick, NJ 08901, USA

**Keywords:** midday napping, older adults, successful aging, Mediterranean region, sleep, MEDIS study

## Abstract

The aim of the present study was to investigate the association between midday napping, sleeping hours, and successful aging among 2564 older (65+ years) individuals living in the insular Mediterranean region. Anthropometric, clinical, and socio-demographic characteristics, dietary habits, and lifestyle parameters were derived through standard procedures, while successful aging was evaluated using the validated Successful Aging Index (SAI; range 0–10). Of the 2564 participants, 74% reported midday napping. The SAI score was 2.9/10 for non-midday nappers vs. 3.5/10 for midday nappers (*p =* 0.001). Midday nappers were more likely to be physically active (*p* = 0.01) and to have higher adherence to the Mediterranean diet (*p* = 0.02) compared to non-midday nappers. In a fully adjusted model, midday nappers had 6.7% higher SAI score compared to the rest (*p* < 0.001), and the effect of midday napping was more prominent among males and participants 80+ years of age. Further analysis indicated a significant U-shaped trend between sleeping hours/day and SAI score *(p* < 0.001), with 8–9 h total of sleep/day, midday napping included, proposed as optimal in achieving the best SAI score. Midday napping seems to be a beneficial habit that should be promoted and encouraged in older people.

## 1. Introduction

Sleep is a basic human need and is essential for good quality of life, good health, and vital for performing well during the day. Inadequate sleep, and even low reductions of sleeping hours for just one week, can lead to pre-diabetic blood sugar levels [1]. Short sleep duration increases the likelihood of coronary heart disease, cardiovascular disease (CVD), stroke, and congestive heart failure [2]. Short sleep or poor sleep quality could also be responsible for immune system disruptions, obesity [3], and some long-term mood disorders such as depression and anxiety [4]. 

It is well established that the process of aging brings many life changes. Older people commonly have at least one sleep problem, as life and sleep patterns change. Stressful events are frequent among older people and may cause disrupted sleep [5]. However, many older people, especially those living in the Mediterranean region, tend to take a short nap in the early afternoon and/or after the midday meal, i.e., midday napping. Midday napping is a common tradition in Mediterranean countries where the weather is usually warm, particularly during day. Midday napping has been recognized as beneficial by sleep experts as it can increase alertness, reduce stress, improve perception, boost memory and mood, and reduce body mass index (BMI) and CVD risk [6,7]. However, since the world’s population is aging rapidly, it is essential to ensure that people age healthily. Healthy or successful aging is a complex phenomenon. It encompasses many factors and situations, i.e., absence of disease and disability, maintenance of cognitive function and physical activity, and engagement in social activities [8]. Many other lifestyle [9] or nutritional [10,11,12] factors commonly seen in the Mediterranean region are already associated with successful aging.

Thus, under the context of the Mediterranean Islands (MEDIS) Study, the aim of the present work was to investigate the association between midday napping, sleeping hours, and successful aging among older individuals living in the insular Mediterranean region. Medical conditions, such as diabetes, obesity, and CVD, develop over long periods of time and result from several factors, such as genetics, poor nutrition, and lack of exercise [13]. Insufficient sleep has also been linked to many health problems, including the ones mentioned above, and is considered an important risk factor. Although scientists have begun to identify the connections between sleep and health, there is still a lack of evidence to determine the role of sleeping behavior in achieving longevity and successful aging.

## 2. Materials and Methods

The MEDIS study [14] is a population-based, observational survey that enrolled older people (*n* = 3325) from 26 Mediterranean islands of 5 countries. Between 2005 and 2018, a random population-based, multistage sampling scheme (i.e., age group, 3 levels (65–75, 75–85, 85+), and 2 sex levels) was applied to voluntarily enroll older males and females from: Malta Republic (*n* = 250), Sardinia (*n* = 60) and Sicily (*n* = 50) in Italy, Mallorca and Menorca (*n* = 111) in Spain, Republic of Cyprus (*n* = 300), Gökçeada (*n* = 55) in Turkey, and the Greek islands of Lesvos (*n* = 142), Samothraki (*n* = 100), Cephalonia (*n* = 115), Crete (*n* = 131), Corfu (*n* = 149), Limnos (*n* = 150), Ikaria (*n* = 76), Syros (*n* = 151), Naxos (*n* = 145), Zakynthos (*n* = 103), Salamina (*n* = 147), Kassos (*n* = 52), Rhodes and Karpathos (*n* = 149), Tinos (*n* = 129), Ai Stratis (*n* = 30), Aigina (*n* = 91), Spetses (*n* = 92), Paros (*n* = 149), Hydra (*n* = 103), as well as the rural region of east Mani (*n* = 295). The participation rate varied according to region, from 75 to 89%. The working dataset with available information about sleep was 2564 males and females. Individuals who resided in assisted-living centers had a clinical history of cardiovascular disease (CVD) or cancer, or had left the island for a considerable period of time during their life (i.e., >5 years), were excluded from participating in the study; these criteria were applied because the study aimed to assess lifestyle patterns that were not a response of individuals modifying how they live due to existing chronic health care conditions or by environmental factors, other than their living milieu. A multidisciplinary group of health scientists (physicians, dietitians, public health nutritionists, and nurses) with experience in field investigation collected all of the required information using a quantitative questionnaire and standard procedures.

### 2.1. Bioethics

The MEDIS study was approved by the Institutional Ethics Board of Harokopio University (16/19-12-2006) and followed the ethical recommendations of the World Medical Association (52nd WMA General Assembly, Edinburgh, Scotland, October 2000). Participants were informed of the study aims and procedures, and all participants provided written informed consent for study participation prior to enrollment.

### 2.2. Measurements

All of the measurements taken in the different study centers were standardized, and the questionnaires were translated into all of the cohorts’ languages following the World Health Organization (WHO) translation guidelines for tool assessment (www.who.int/substance_abuse/research_tools/translation/en/).

#### 2.2.1. Sociodemographic and Lifestyle Characteristics 

The socio-demographic characteristics studied included age (years), sex (male/female), marital status (single/married/divorced/widowed), region, and educational and financial status (years of school and average income). Data on hours of sleep/day, midday napping (yes/no, exact duration of napping was not recorded), and frequency (times/week) of napping during midday [15] were recorded. Participants who reported midday napping for > 5 days per week were defined as “midday nappers”. Smoking status was evaluated following standard procedures for observational studies. Particularly, “Current” smokers were defined as those who smoked at least one cigarette or any type of tobacco per day at the time of the interview. “Former” smokers were defined as those who previously smoked but had ceased smoking within the previous year. Current and former smokers were combined as “Ever” smokers, while the remaining participants were defined as “Nonsmokers”. 

#### 2.2.2. Physical Activity Levels Physical 

Activity was evaluated in metabolic equivalent (MET) minutes per week, using the shortened, translated, and validated in the Greek version of the self-reported International Physical Activity Questionnaire (IPAQ) [16]. Those who reported at least 3 MET-minutes per week were classified as “Physically active”, while others were defined as “Physically inactive”.

#### 2.2.3. Anthropometric and Clinical Characteristics

Weight and height were measured using standard procedures to attain the volunteer’s BMI (kg/m^2^). Overweight was defined as BMI between 25.0 and 29.9 kg/m^2^, while obesity was defined as BMI > 29.9 kg/m^2^. Type 2 diabetes mellitus (T2DM) was determined by measuring fasting plasma glucose and in accordance with the American Diabetes Association diagnostic criteria (fasting blood glucose >126 mg/dL or use of anti-diabetic medication). Participants who had blood pressure levels >140/90 mmHg or who were administered antihypertensive medications were classified as hypertensive. Fasting blood lipids levels were also recorded. Hypercholesterolemia was defined as total serum cholesterol levels >200 mg/dL or the use of lipid-lowering agents according to the National Cholesterol Education Program Adult Treatment Panel III guidelines [17]. The coefficient of variation for the blood measurements was less than 5%. 

#### 2.2.4. Cognitive Function Assessment Cognitive

Function was evaluated using the Mini-Mental State Examination (MMSE), culturally adopted for the Greek population, with a range of 0–30 [18,19].

#### 2.2.5. Dietary Habits Assessment

Dietary habits were assessed through a semi-quantitative, validated, and reproducible food frequency questionnaire (FFQ) [20]. Consumption of various food groups and beverages (i.e., meat and meat products, poultry, fish, milk and other dairy products, fruits, vegetables, greens and salads, legumes, cereals, pasta, soft drinks, alcohol, coffee, and tea) was measured, in times of weekly consumption (never, rare, 2–3 times/month, 1–2 times/week, 3–5 times per week, and daily). Particularly for tea and coffee consumption, all participants were asked if they consume tea or coffee and the frequency, they consume a cup (of 150 mL) within a week (i.e., never, <1 cup/week, 1–2 cups/day, 3–5 cups/day, >5 cups/day). Moreover, to evaluate the level of adherence to the Mediterranean diet, a previously developed and validated MedDietScore with a theoretical range of 0–55 was used, with higher values indicating greater adherence [21].

#### 2.2.6. Successful Aging Index 

A successful aging index (SAI), ranging from 0 to 10, which has been previously developed and validated [15], using 10 attributes that reflect and are associated with the aging process, was applied for assessing successful aging. The index encompasses health-related social, lifestyle, and clinical factors, including education, financial status, physical activity, BMI, depression, participation in social activities with friends and family, number of yearly excursions, total number of clinical CVD risk factors (i.e., history of hypertension, diabetes, hypercholesterolemia, obesity), and level of adherence to the Mediterranean diet [14].

### 2.3. Statistical Analysis

Continuous variables are presented as mean ± standard deviation (SD), and categorical variables as frequencies. Associations between categorical variables and midday napping (i.e., midday napping or no midday napping) were evaluated using the chi-squared test, while associations between continuous variables and midday napping were evaluated with Pearson’s *t*-test or analysis of variance (ANOVA). Linear regression was used to evaluate the association between midday napping (independent variable) with successful aging (outcome) after adjusting for various participants’ characteristics (i.e., age, sex, smoking habits, tea and coffee consumption). Similar analyses were conducted, stratified by sex (female, male), age-group (< or >80 years), and lifestyle-related sleep behaviors. Results are presented as beta coefficients, standard error, and *p*-value. The linearity of models’ fitting was tested through the scatter plots of standardized residuals against fitted values; normality of the continuous variables (i.e., sleeping hours/d), as well regression residuals, were evaluated through P–P (Probability-Probability) plots; dependency was tested using the Durbin–Watson test and homoscedasticity using the variance inflation index (a value <4 suggests lack of heteroscedasticity). Logistic regression analysis was also used to evaluate the association between midday napping vs. no midday napping and the likelihood of having above the median successful aging index (outcome). Results from logistic regression analysis are presented as odds ratio (OR) and the corresponding 95% confidence interval. Curve estimation using linear and non-linear regression models was applied to evaluate the association between hours of sleep per day and SAI score. The results are presented as *b*-coefficients of the model that was fitted to the data points and the corresponding quadratic line (with ±1 SD confidence bands). Moreover, LOWESS (locally weighted scatterplot smoothing) was also applied to illustrate the association between total sleeping hours and successful aging index. STATA (M. Psarros & Associates, Sparti, Greece) software version 15 was used for all calculations.

## 3. Results

### 3.1. Midday Napping and Successful Aging

Of the 2564 participants of the MEDIS study, 74% reported midday napping. Midday nappers were 56% males and 44% of females (*p =* 0.02). Table 1 illustrates participants’ characteristics according to midday napping behavior and sex. Overall, compared to non-midday nappers, midday nappers had a 20% higher level of SAI score (3.5 vs. 2.9, *p =* 0.001), were more likely to be physically active (*p* = 0.01), and to have higher adherence to the Mediterranean diet (*p =* 0.02). 

As residual confounding may always exist in observational studies, Table 2 presents results from multi-adjusted, nested linear regression models that evaluated the association of midday napping on successful aging (outcome) among MEDIS study participants, after taking into account various potential confounders. It was found that compared to non-midday nappers, participants who reported midday napping had 0.577 units (or 5.7%) higher SAI score (*p* < 0.001), after adjusting only for age. This association remained significant, and became even stronger after adjusting for several lifestyle factors related to sleep, such as sex, smoking habits, coffee and tea consumption. Specifically, in the fully adjusted model 5, midday nappers had 6.7% higher SAI score compared to the rest of the participants. When cognitive function was included in the models, the results did not show statistical significance (*p* > 0.05).

Further analysis stratified by sex showed that in the age-adjusted model 1, female midday nappers had a 5.9% (±1.4%) higher SAI score compared to non-midday nappers (*p* = 0.008), whereas male midday nappers had an increase of 4.9% ± 1.4% in SAI score (*p* = 0.01) (for sex comparisons, *p* < 0.001). When smoking habits, as well as coffee and tea consumption, were included in the models, the aforementioned association became stronger, but the effect of midday napping among males became more prominent as compared to females. In particular, female midday nappers presented an increase of 6.2% ± 1.3% in SAI score compared to non-midday nappers (*p =* 0.002), while male midday nappers had an increase of 7.4% in SAI score (*p <* 0.001) (for sex comparisons, *p <* 0.001).

**Figure 1:**
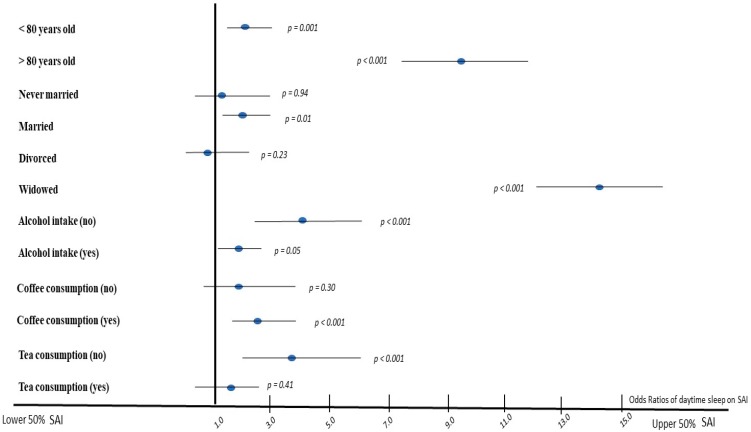
Results from logistic regression models to evaluate the association between midday nappping vs. no midday napping and the likelihood of having above the median successful aging index. Subgroup logistic regression analysis has been adjusted for age, sex, alcohol intake, and coffee and tea consumption. Results from the subgroup logistic regression analysis are presented as odds ratios and the corresponding 95% confidence interval.

In Figure 1, effect size measures (i.e., OR) from multi-adjusted logistic regression models are illustrated that evaluated the association between midday napping behaviors and the likelihood of having a “high” SAI score (i.e., above the median, upper 50% of SAI score), after taking into account the effect of age, sex, coffee and tea drinking, and alcohol consumption. As it can be drawn from Figure 1, older old (i.e., over 80 years) midday nappers had 9-times higher odds of having “high” SAI scores (*p* < 0.001) compared to their no midday napping counterparts; the effect of midday napping among younger individuals was less prominent (i.e., 1.9-times higher odds of having “high” SAI scores, *p* < 0.001). Concerning the effect of marital status, widowed midday nappers had 14.8-times higher odds of having “high” SAI scores *(p <* 0.001) compared to their no midday napping counterparts; and the effect of midday napping among married individuals was less prominent (i.e., 1.8-times higher odds of having “high” SAI scores, *p* = 0.01), whereas no significant association was observed for midday nappers who reported as “never married” or “divorced” (both *p* > 0.05).

Further analysis was applied to assess the role of some lifestyle-related sleep behaviors, like coffee and tea drinking and alcohol consumption. It was found that compared to non-midday nappers, midday nappers who did not consume tea had 3.9-times higher odds of being in the upper 50% of SAI score levels *(p <* 0.001). However, no significant association was observed for midday nappers who reported as tea drinkers *(p =* 0.40). Moreover, midday nappers who also reported as coffee drinkers had 2.7-times higher odds of having a “high” SAI score compared to non-midday nappers, whereas no significant association of midday napping on SAI was observed among non-coffee drinkers (*p =* 0.30). Regarding the effect of alcohol consumption, non-alcohol drinkers who also reported midday napping had 3.9-times higher odds of having a “high” SAI score, compared to non-midday nappers *(p <* 0.001), whereas, similar associations were observed for midday nappers who were alcohol drinkers, i.e., had 1.9-times higher odds of having “high” SAI scores compared to non-midday nappers *(p =* 0.05).

### 3.2. Sleeping Hours and Successful Aging

**Figure 2:**
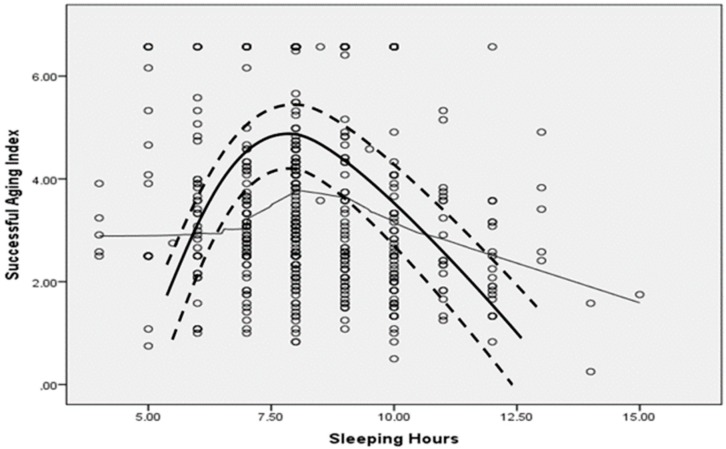
Scatter plot with LOWESS line and quadratic line (thick line, with confidence bands at ±1 SD) corresponding to the relationship between sleeping hours (X-axis) and successful aging index (SAI) (Y-axis).

The mean (SD, median) sleeping hours were 8.2 ± 1.7, 8 h in males and 8.1 ± 1.6, 8 h in females (*p* = 0.70). Mean SAI values by quartile of sleeping hours per day were: <6 h, 3.0 ± 1.1 vs. 6–9 h, 3.2 ± 1.1 vs. 9–10 h, 2.8 ± 1.1 vs. >10 h, 2.5 ± 1.0 (*p <* 0.001). Further analysis was applied to evaluate the association between sleeping hours and SAI score. There is indication that a significant quadratic (i.e., inverse U-shape) trend was observed between sleeping hours per day and SAI score in males (coefficients of the 2nd order polynomial line, *b*_1_, *b*_2_: 0.18, 0.01, *p <* 0.001) and in females (*b*_1_, *b*_2_: 0.36, 0.04, *p <* 0.001). Figure 2 presents scatter plots with LOWESS and quadratic lines corresponding to the relationship between sleeping hours (X-axis) and successful aging index (Y-axis) among MEDIS study participants. No alterations in the aforementioned findings were observed when the analysis was focused on males or females, older or younger participants, coffee, tea or alcohol drinkers or not (for all interactions, *p* > 0.5). Threshold analysis showed that the optimal total sleeping hours to achieve better SAI levels were 8.5 h per day.

**Figure 3:**
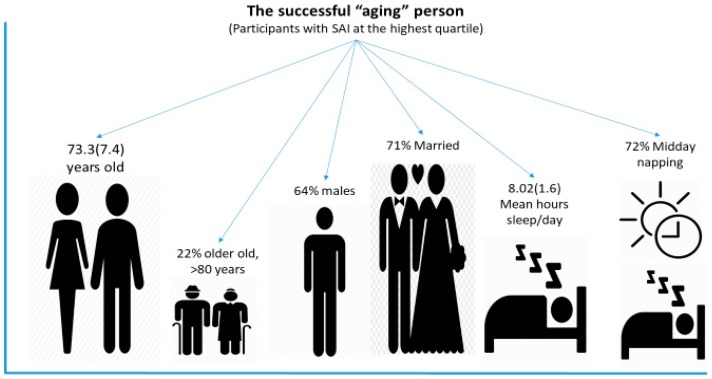
The characteristics of the successful “aging” person. In this graph, the analysis was focused on participants belonging in the highest tertile of the SAI (i.e., >3.7); mean hours of sleeping/day, percent of midday napping, as well as age, sex, and percent of older old individuals (i.e., >80 years) are illustrated.

Then, SAI was categorized into quartiles (i.e., <1.6, 1.6–2.6, 2.6–3.7, and >3.7) and the main characteristics of the participants were explored focused on the highest tertile (i.e., over 3.7). It was observed that participants belonging in the highest tertile of SAI were mainly males (i.e., 64%), 22% of them were over 80 years of age, 71% were married (17% widowers/widows), and 72% were midday nappers. The total mean hours of sleeping per day was 8.02 ± 1.6. In Figure 3, the characteristics of the successful “aging” person are illustrated.

## 4. Discussion

The present study aimed to evaluate the association between midday napping, sleeping hours (as a duration of sleep), and successful aging among older individuals living in the Mediterranean region. Our results, based on a large-scale, multi-center epidemiological study, revealed that midday napping was positively associated with higher levels of successful aging. Stronger associations were observed in male midday nappers compared to females, in non-tea drinkers, but also among coffee drinkers. Alcohol consumption did not have any synergistic effect with midday napping on successful aging level of the participants. Moreover, age-group analyses showed that among >80-year-old, those who reported midday napping had almost 9-times higher odds of achieving “high” successful aging levels, as compared to non-midday nappers; this effect was less prominent among younger participants. Regarding duration of sleep, it was observed that the optimal hours of sleep in order to achieve the best possible successful aging level was 8.5 h/day. Those 8.5 h need not be all at night and there is an indication that it is more beneficial if they are not slept all together. Despite the limitations of the present cross-sectional, observational study, the presented findings revealed an important role of sleep behaviors, i.e., midday napping, on successful aging in a Mediterranean population. 

Mediterranean people were traditionally known for their low levels of heart disease. However, nowadays, the prevalence of heart disease in the Mediterranean region has increased, and could be attributed to many factors. One of the main factors is that Mediterranean eating habits, traditionally high in plant-based foods, are slowly deteriorating, while Western eating and lifestyle habits are becoming more prevalent [22]. Meanwhile, midday napping is a feature of normal life in Mediterranean and Latin American countries. Like many traditional lifestyle habits around the world, napping in the day has become less common because of globalization, new employment regimes, and a busier, more stressful lifestyle, especially in countries and regions with economic instability [23]. An interesting observation, however, is that midday nappers had better nutritional habits, i.e., they are better adherents of the Mediterranean diet compared to non-midday nappers. Therefore, it is suggested that the adherence to the Mediterranean diet may help to improve the quality of sleep or vice versa in older people [24], supporting a potential idea that lower adherence to traditional habits may lead to worse health and quality of life. 

Sleep is a vital indicator of overall health and well-being, and it is estimated that humans spend up to 33% of their life asleep. Sleep habits and needs vary across ages and sex and are impacted by lifestyle behaviors and health [25]. Previous research has shown that sleep latency increases with age in females, whereas sleep efficiency decreases in both females and males [26]. Females usually report worse quality and disrupted sleep across the ages. This could be attributed to sex steroids in sleep modulation [27]. In the present study, it was found that midday napping affects both males and females positively, but there were still some differences between them. This is further supported by Madrid-Valero et al [28], who, after examining 2144 individuals from Spain, found that not only age is inversely associated with quality of sleep, but also that quality of sleep varies by sex, with females following a steady worsening in sleep quality [28]. Additionally, another study examining the association between circadian alignment and BMI, body fat, and obesity-related behaviors revealed that circadian alignment was associated with dietary intake, while a sex difference between the previously mentioned association was suggested [29]. 

Married people usually sleep together with their spouse, while divorced or widowed people sleep alone. There is evidence suggesting that “sleep divorce”, i.e., sleeping in a separate bed to your partner, may benefit both health and quality of life. According to a survey conducted in 2000 Americans, almost half of the respondents reported that they prefer to sleep alone, while 19% blamed their partner for their poor sleep [30]. A cross-sectional study of 405 couples aged between 51 and 94 years in the United States also identified a negative impact of spousal sleep problems with their partner’s health and well-being [31]. Widowhood and divorce are usually distressing and disturbing events in life, but the level of sleep disturbance may vary. These could be potential reasons why widowed midday nappers presented higher levels of SAI compared to non-midday napping counterparts, as well as married or never-married individuals, or even those divorced. 

Sleep is usually regulated by two body systems, i.e., sleep/wake homeostasis and the circadian rhythm [32]. However, stimulants such as coffee, tea, energy and soft drinks, or even alcohol may interfere with the circadian rhythm [33]. Alcohol has a soporific effect; as blood alcohol levels decrease, the rate of arousal increases [34]. In addition, caffeine has psychoactive effects, and may reduce total sleep time and sleep quality if consumed later in the day [35]. However, in the present study, it was found that midday napping was associated with higher levels of successful aging among coffee and no-tea drinkers. In a recent study by Spadola et al., [36], examining the night-to-night association of evening use of alcohol and caffeine among others with sleep behaviors, it was observed that alcohol disrupted sleep, but caffeine seemed to have no effect [36]. Moreover, results from the MEDIS study have previously shown that tea consumption and, more specifically, green but not black tea consumption may enhance successful aging [37]. 

The fact that middle-aged individuals spend many hours working and their sleep duration is usually short has been associated with a variety of health consequences in older age, such as decreased physical functioning [28], a component of successful aging. According to the National Sleep Foundation, sleep duration recommendations for older adults, in order to avoid serious health problems or maintain health and well-being, range from 7 to 8 h/day [38]. This was supported by a study of older Chinese people who slept less than 6 h or more than 7 h/day, and had lower odds of successful aging [39]. In the present study, however, it was found that 8.5 h (not necessarily slept all together) seems to be the optimal sleeping hours for older Mediterranean people to achieve successful aging. As Chaput et al. [40] suggest, sleep needs are determined by several genetic, environmental and behavioral factors, and sleep duration should be personalized based on these factors [40]. It is also suggested that longer sleep has been associated with mental health problems in older people [41]; however, people living in the Mediterranean region traditionally practice napping in the day and therefore increase their overall duration of sleep without necessarily acquiring health-related problems. In general, older people tend to go to sleep earlier and wake earlier than younger adults for a number of different reasons, such as insomnia and sleep apnea, which are the most common sleep problems among older people [42]. A survey in older African-American people from disadvantaged financial areas of South Los Angeles revealed that insomnia symptoms co-exist with many mental, physical, and financial challenges and that sleep could be one of the ways to cope with these challenges [43]. Sleep apnea can also cause other problems as well, such as memory loss, high blood pressure, or even stroke [44], and all of these problems affect the quality of life of older people, their sleep duration, and therefore their course of aging. 

Sleep is essential for health and quality of life. However, sleep is actually a complex phenomenon, similarly to successful aging. For that reason, it is vital to understand the underlying causes of sleep problems and to identify and overcome age-related sleep problems to promote the quality of sleep and, therefore, the quality of everyday life. Adequate sleep may improve mental health so as to maintain mental functionality and physical performance throughout the day, and may help to avoid chronic conditions such as depression and CVD. Thus, midday napping, as well as restful sleep during the night hours, should be highly promoted to increase odds of successful aging and mental and physical functionality. Nowadays, an increasing number of people tend to spend time exposed to blue light watching television or using a computer, which can potentially cause sleep deprivation and consequently affect their quality of sleep [45]. Electronic devices which emit blue light are commonly used in the hours before sleep, decreasing melatonin and increasing alertness [46]. As such, the role of frequent use of technology in the evening hours on sleep quality and successful aging represents an important area of future research.

To the best of our knowledge, this is one of the first studies to evaluate the associations of midday napping on successful aging in older people living in the Mediterranean region. However, the conclusion of the present study should be considered under the existing limitations. The observational nature of the cross-sectional design does not allow for causal associations to be drawn. Moreover, blood evaluation and genetic evaluation of biological age were not feasible. Sleeping behaviors and hours of sleep were not evaluated via actigraphy, but were based on questionnaires, and thus recall bias may exist. Finally, successful aging was not evaluated through cognitive behavior, neither was mobility nor physical function of the participants. These factors often characterize successful aging, along with low probability of disease and active engagement with life, and this may constitute another limitation. However, the successful aging index, used in this work, has already been validated [14].

## 5. Conclusions

Sleep is an essential component of a healthy lifestyle, with both short and long duration of sleep associated with various morbidities and higher mortality. However, the effect of midday napping has rarely been investigated, especially regarding the successful aging process. As reported here, midday napping was independently associated with higher levels of successful aging, whereas 8.5 h of sleep per day in total, not necessarily slept all together, seems to be the optimal duration for achieving the best successful aging level. These findings deserve further attention from a public health perspective, since older people, a rapidly growing population, have not been well studied or understood regarding the relationship between successful aging, sleep deprivation, and sleep disorders. 

## Figures and Tables

**Table 1:** Socio-demographic, lifestyle, and clinical characteristics of the MEDIS study’s participants based on midday napping.

	Non-Midday Nappers			Midday Nappers					
	All	Females	Males	All	Females	Males	*p*	*p* ^1^	*p* ^2^
Males *n* (%)	318 (47)	-	-	1065 (56)	-	-	0.02	-	-
Age (years)	72 ± 6.7	72 ± 6.3	73 ± 7.1	73 ± 7.3	73 ± 7.1	74 ± 7.5	0.37	0.48	0.69
Smoking, %yes	20	8	34	17	5	26	0.27	0.26	0.12
Physically active, %active	26	21	31	18	14	21	0.01	0.09	0.04
BMI (kg/m^2^)	28 ± 4.5	28 ± 5.3	28 ± 3.5	28 ± 4.2	29 ± 4.7	28 ± 3.8	0.72	0.24	0.59
Coffee intake, %yes	87	86	88	88	84	91	0.63	0.70	0.36
Tea intake %yes	43	53	32	57	59	54	0.001	0.25	<0.001
Alcohol intake %yes	44	17	75	44	17	65	0.95	0.97	0.06
MedDietScore (0–55)	32 ± 5.0	32 ± 5.1	34 ± 4.5	34 ± 4.0	34 ± 3.8	34 ± 4.1	0.02	0.002	0.94
Hypertension, %yes	55	55	55	65	71	59	0.002	0.01	0.04
Diabetes, %yes	21	18	23	26	25	26	0.14	0.13	0.59
Hypercholesterolemia, %yes	50	51	48	45	47	44	0.28	0.48	0.47
MMSE (0–30)	24 ± 4	24 ± 4	25 ± 4	24 ± 4	24 ± 3	25 ± 4	0.97	0.43	0.72
SAI (0–10)	2.9 ± 1.3	2.5 ± 1.3	3.3 ± 1.3	3.5 ± 1.9	3.2 ± 2.1	3.8 ± 1.8	0.001	0.01	0.01

Values are presented as percent (%) or mean ± standard deviation. *p*: *p*-values for total sample derived from Pearson’s *t*-test for continuous variables or the chi-square test for the categorical variables. *p*^1^: For female non-midday nappers vs. female midday nappers. *p*^2^: For male non-midday nappers vs. male midday nappers. BMI: Body mass index. SAI: Successful aging index.

**Table 2:** Results from linear regression models that evaluated the association of the midday napping on successful aging (independent outcome) after adjusting for age, sex, smoking, coffee and tea consumption, among MEDIS study participants.

	b ± SE For Midday Napping (Yes/No)	95% CI	*p*
**Model 1:** Age	0.577 ± 0.142	0.298–0.856	<0.001
**Model 2:** Model 1 + sex	0.519 ± 0.140	0.244–0.795	<0.001
**Model 3:** Model 2 + smoking	0.515 ± 0.141	0.238–0.792	<0.001
**Model 4:** Model 3 + coffee consumption	0.521 ± 0.141	0.244–0.797	<0.001
**Model 5:** Model 4 + tea consumption	0.667 ± 0.137	0.398–0.936	<0.001

Results are presented as unstandardized *b*-coefficients for midday napping (yes/no), standard error (SE), their 95% confidence interval (CI), and *p*-value.
